# Effects of ω3- and ω6-Polyunsaturated Fatty Acids on RANKL-Induced Osteoclast Differentiation of RAW264.7 Cells: A Comparative *in Vitro* Study

**DOI:** 10.3390/nu6072584

**Published:** 2014-07-09

**Authors:** Jan C. A. Boeyens, Vishwa Deepak, Wei-Hang Chua, Marlena C. Kruger, Annie M. Joubert, Magdalena Coetzee

**Affiliations:** 1 Department of Physiology, University of Pretoria, Pretoria, Private Bag X323, Arcadia 0007, South Africa; E-Mails: christob@oncology-sa.co.za (J.C.A.B.); vishwa.deepak@up.ac.za (V.D.); annie.joubert@up.ac.za (A.M.J.); 2 Institute of Food, Nutrition and Human Health, Massey University, Palmerston North 4442, New Zealand; E-Mails: w.h.chua@massey.ac.nz (W.-H.C.); m.c.kruger@massey.ac.nz (M.C.K.); 3 Department of Human Nutrition and Associate of the Institute for Food, Nutrition and Well-being, University of Pretoria, Pretoria 0002, South Africa

**Keywords:** osteoclasts, polyunsaturated fatty acids, bone resorption, arachidonic acid, gamma-linolenic acid, eicosapentaenoic acid, docosahexaenoic acid, RAW264.7 macrophages

## Abstract

Polyunsaturated fatty acids (PUFAs) have been reported to have an anabolic effect on bone *in vivo*, but comparative studies to identify inhibitors of osteoclast formation amongst ω3- and ω6-PUFAs are still lacking. Here we assessed the effects of the ω3-PUFAs, eicosapentaenoic acid (EPA) and docosahexaenoic acid (DHA) and the ω6-PUFAs, arachidonic acid (AA) and γ-linolenic acid (GLA) on a RAW264.7 osteoclast differentiation model. The effects of PUFAs on RANKL-induced osteoclast formation were evaluated by counting tartrate resistant acid phosphatase (TRAP)-positive multinucleated cells. PUFAs significantly inhibited RANKL-induced osteoclast formation in a dose-dependent manner with AA- and DHA-mediated inhibition being the strongest. Furthermore, RANKL-induced mRNA- and protein expression of the key osteoclastogenic genes cathepsin K and TRAP were inhibited by AA and more potently by DHA. Owing to the attenuated osteoclastogenesis by DHA and AA, actin ring formation and bone resorptive activity of these cells as evaluated on bone-mimetic plates were severely compromised. Hence, of the tested PUFAs, AA and DHA were found to be the most effective in inhibiting RANKL-induced osteoclast formation with the latter providing the strongest inhibitory effects. Collectively, the data indicates that these PUFAs may play an important role in regulating bone diseases characterized by excessive osteoclast activity.

## 1. Introduction

A balanced and nutritious diet is critical for sustaining good health. Epidemiological and clinical studies support the notion that adequate nutrient intake aids in the prevention of chronic diseases such as osteoporosis and osteoarthritis [1]. Most bone-related diseases are characterized by excessive bone loss due to increased bone resorption and reduced bone formation. During old age, the process of bone remodelling coupled by the action of osteoclasts and osteoblasts often becomes unbalanced to favour bone resorption leading to a gradual decline in bone mineral density [2].

Osteoclasts are giant multinucleated cells formed by the fusion of mononuclear stem cells that are common to lymphocytes, granulocytes and mononuclear phagocytes [3]. Although the function of osteoclasts and osteoblasts are counteractive, the latter are essential for bone resorption as they secrete factors that are necessary for osteoclast differentiation. Receptor activator of nuclear factor-kappa B ligand (RANKL), a member of the tumour necrosis factor (TNF) superfamily, is one such molecule that is expressed in both membrane-bound and soluble form by osteoblasts. The binding of RANKL to its receptor RANK on precursor cells is an essential step in the activation of osteoclastogenesis that involves the fusion of mononuclear osteoclast-precursors into mature multinucleated osteoclasts [3]. Mature osteoclasts express tartrate resistant acid phosphatase (TRAP), an enzyme widely used as a mature osteoclast marker and essential for collagen breakdown and bone matrix resorption [4]. Bone resorption occurs within the sealing zone that is formed by an actin ring structure containing podosomes that anchors osteoclasts to the bone surface and is characterized by dissipation and degradation of two components of the bone matrix, the inorganic mineral crystalline hydroxyapatite and the organic collagen rich matrix. Proteases, namely cathepsin K, are responsible for the degradation of the organic bone matrix, releasing collagen fragments [5].

ω6 (*n*-6) and ω3 (*n*-3) fatty acids are the two principle families of polyunsaturated fatty acids (PUFAs). Linoleic acid (LA, 18:2*n*-6; precursor of ω6 PUFAs) and α-linolenic acid (ALA, 18:3*n*-3; precursor of ω3 PUFAs) are the simplest of each of these two families respectively and are considered essential fatty acids (EFA) since they cannot be synthesised by mammals [6]. Linoleic acid can be converted stepwise into PUFAs such as γ-linolenic acid (GLA) by ∆^6^ desaturase and is elongated to form dihomo-γ-linolenic acid (DGLA) that is further converted into arachidonic acid (AA) by the enzyme ∆^5^ desaturase [7]. By a similar set of reactions that are catalysed by the same group of enzymes, α-linolenic acid can be converted to eicosapentaenoic acid (EPA) and docosahexaenoic acid (DHA) [8,9]. Although EPA (20:5*n*-3) and DHA (22:6*n*-3) are considered non-essential fatty acids due to the fact that they can be synthesised in the body from ALA, this conversion is not efficient enough in humans to meet EPA and DHA requirements [9,10]. These fatty acids need to be obtained from the diet to have beneficial health effects. ω3-PUFAs are found in abundant quantities in plants, eggs, fish, nuts and berries. Dietary sources of ω6-PUFAs include vegetable seeds and oils such as those from safflower, sunflowers, soybeans and corn [11,12].

Evidence presented over the past 20 years has shown that long chain polyunsaturated fatty acids (LCPUFAs) are beneficial to bone health [13]. Several studies have confirmed the bioactivity of PUFAs both in humans and animals and have shown key roles of ω3- and ω6-fatty acids in delaying bone degradation, improving structural and mechanical properties of cortical bone, and improving the bone mineral density of elderly women with confirmed osteoporosis [14]. The large amount of research conducted on the bone protective effects of PUFAs report data for individual fatty acids neglecting the requirement of a comparative study. To accurately define the roles of PUFAs in regulating bone metabolism it is essential to design a study where most of the PUFAs could be studied in similar experimental conditions giving more precise and useful results.

The murine macrophage RAW264.7 is an osteoclast precursor cell line that endogenously expresses the c-fms receptor for macrophage-colony stimulating factor (M-CSF) as well as M-CSF itself and therefore undergoes *in vitro* differentiation and forms bone resorbing multinucleated osteoclasts when stimulated with RANKL ([15] and references therein). Since, RANKL is the primary osteoclast differentiation signal that elicits the formation of osteoclast by binding to RANK and thereby activating downstream signalling events necessary for osteoclast formation, inhibition of the RANKL/RANK pathway is considered as an effective approach to attenuate osteoclastogenesis and bone resorption. In this study, the ω3-PUFAs, EPA and DHA, and the ω6-PUFAs, AA and GLA were employed to comparatively assess the effects of these fatty acids on RANKL-induced osteoclast differentiation of RAW264.7 murine macrophages.

## 2. Experimental Section

### 2.1. Reagents and Materials

Dulbecco’s Modified Eagle Medium (DMEM), heat inactivated fetal bovine serum (FBS) and antibiotic-antimycotic solutions (#15240) were supplied by GIBCO (Invitrogen Corp., Victoria, Australia). Fatty acids (AA, GLA, EPA, DHA), protease cocktail inhibitors (#P2714), TRI^®^ reagent (#T9424) and Leukocyte Acid Phosphatase (TRAP) kits (#387A-KT) were obtained from Sigma-Aldrich Inc. (St. Louis, MO, USA). RANKL (#462-TEC) was purchased from Research and Diagnostic Systems (R & D Systems, Minneapolis, MN, USA). Corning Osteo Assay Surface plates were obtained from Corning Life Sciences (New York, NY, USA) and bovine cortical bone slices (DT-1BON1000-96) were supplied by Immunodiagnostic Systems Ltd. (Boldon, UK). Cell extraction buffer (#FNN0011), NuPAGE Novex Bis-Tris precasted polyacrylamide gel (#NP0322BOX), iBlot dry blotting system (#IB4010-01), iBlot Western Detection Chromogenic Kit (#IB7410-02) were acquired from Life Technologies (Carlsbad, CA, USA). The bicinchoninic acid (BCA) protein assay kit (#23227) was supplied by Thermo Scientific (Rockford, IL, USA). M-MuLV reverse transcriptase (#M0253S) was purchased from New England Biolabs (Hitchin, UK). KAPA2G Robust HotStart ReadyMix (#KK5701) was bought from Kapa Biosystems (Cape Town, South Africa). Rabbit polyclonal antibodies against GAPDH (#37168), MMP-9 (#38898), cathepsin K (#19027), TRAP (#96372) were procured from Abcam (Cambridge, MA, USA) and the goat-anti-rabbit alkaline-phosphatase-conjugated secondary antibody was supplied as a component with iBlot Western Detection Chromogenic Kit.

### 2.2. Cell Culture and Maintenance

RAW264.7 cells (#TIB-71) were purchased from the American Type Culture Collection (ATCC, Rockville, MD, USA) and maintained in DMEM with 10% FBS. All media were supplemented with penicillin (100 U·mL^−1^), streptomycin (100 μg·mL^−1^) and fungizone (0.25 μg·mL^−1^). Cells were incubated at 37 °C in 5% CO_2_ in humidified air. Fatty acids were prepared in ethanol and 100 mg·mL^−1^ aliquots were stored in the dark at −80 °C until required. The final ethanol concentration in the culture medium did not exceed 0.01% (v/v). All of the cell culture experiments including tested PUFAs as well as control with RANKL, were vehicle treated (ethanol, 0.01% v/v). Cells were exposed to RANKL alone or RANKL in combination with various PUFAs at the same time throughout the experiments.

### 2.3. Tartrate Resistant Acid Phosphatase (TRAP)-Positive Cell Staining

RAW264.7 cells suspended in DMEM containing 10% FBS were seeded into sterile 24-well culture plates at a density of 1.5 × 10^4^ cells per well. Cells were simultaneously exposed to fatty acids at 5–20 μg·mL^−1^ and 15 ng·mL^−1^ RANKL. Effective fatty acids and RANKL concentrations used in this study for TRAP staining, immunoblots and PCR were determined by titration in our laboratory. Cells seeded into medium containing ethanol (0.01%, v/v) served as vehicle control. Cell culture media and factors were exchanged after three days. After a total of five days, cells were fixed and stained for TRAP using the Leukocyte Acid Phosphatase staining kit followed by counterstaining with haematoxylin according to the manufacturer’s instructions. TRAP-positive multinucleated cells containing more than 3 nuclei were counted as osteoclasts. Photomicrographs were taken with a Zeiss Axiocam MRc5 camera attached to a Zeiss Axiovert 200 microscope (Zeiss, Oberkochen, Germany).

### 2.4. Fluorescent Microscopy

Fluorescent microscopy was employed to observe actin ring formation in osteoclasts differentiated from RAW264.7 cells. Cells were suspended in DMEM containing 10% FBS and seeded into sterile 6-well culture plates at a density of 1 × 10^5^ cells per well and were exposed to 30 ng·mL^−1^ RANKL with 20 μg·mL^−1^ of AA or DHA at the same time. Effective RANKL concentrations for microscopy and resorption experiments were established by titration in our laboratory. Cells seeded into medium containing ethanol (0.01%, v/v) served as a vehicle control. After five days of differentiation with a change of medium and factors after 3 days, cells were washed with PBS and fixed with a 3.7% formaldehyde solution. Cells were permeabilized with 0.2% Triton X-100 in PBS for 10 min and were stained with 50 μg·mL^−1^ fluorescent phalloidin conjugate solution (Sigma-Aldrich) for 40 min at room temperature. After staining, cells were washed with PBS and the nuclei were stained with bisbenzimide (Hoechst 33342, Sigma-Aldrich) and rinsed with PBS. Actin rings and nuclei were visualised fluorescently using a Zeiss inverted Axiovert CFL40 microscope attached to a Zeiss Axiovert MRm monochrome camera using the appropriate filter sets: Hoechst (Excitation: 352 nm, Emission: 455 nm); Phalloidin (Excitation: 502 nm, Emission: 525 nm) (Zeiss). Three independent experiments were conducted in duplicate.

### 2.5. Scanning Electron Microscopy

Scanning electron microscopy (SEM) was used to determine the surface features of cells cultured on bovine cortical bone slices. RAW264.7 cells (1 × 10^4^ cells per well) were suspended in DMEM containing 10% FBS and seeded into a 24-well culture plate containing three bone slices per well. Cells were seeded in the presence of 30 ng·mL^−1^ RANKL and were simultaneously exposed to AA or DHA at a concentration of 20 μg·mL^−1^; ethanol (0.01%, vehicle, v/v) was used as control. Media and factors were replaced on days 3 and 6. After 9 days, bone slices were removed and the cells were fixed and prepared for SEM as described earlier [16]. The bone slices with cells were mounted on a stub, and sputter coated with gold. Samples were visualised using a JEOL 840 scanning electron microscope (JEOL, Tokyo Japan).

### 2.6. Resorption Assay on Bone Mimetic Plates

RAW264.7 cells (1.5 × 10^4^ cells per well) were suspended in DMEM containing 10% FBS and seeded onto Corning Osteo Assay 24-well plates. Cells were seeded in the presence of 30 ng·mL^−1^ RANKL and AA or DHA at a concentration of 20 μg·mL^−1^. Media and factors were replaced on days 3 and 6. After 9 days, cells were washed off the bottom of the plates with a 5% hypochlorite solution. Resorbed areas on the plates were captured with a Zeiss Axiocam ERc5 microscope attached to a Zeiss Axiocam MRc5 camera and analysed with ImageJ analysis software [17].

### 2.7. Western Blot Analysis

RAW264.7 cells (4 × 10^4^ cells per well) were differentiated in the presence of RANKL (15 ng·mL^−1^) and PUFAs (20 μg·mL^−1^) for 5 days in a 24-well plate. Cell culture media and factors were exchanged after three days. At the end of the culture, cells were lysed in ice-cold cell extraction buffer supplemented with protease cocktail inhibitors for 30 min on ice, with vortexing at 10 min intervals. Resultant cell lysates were centrifuged at 15,000× *g* for 30 min at 4 °C and the resultant supernatant was used for further experiments. Purified proteins were quantified using a BCA protein assay kit as per manufacturer’s directions. Twenty five micrograms of protein lysates were loaded onto each lane and resolved on a 4%–12% NuPAGE Novex Bis-Tris precasted polyacrylamide gel and electrotransferred to PVDF membranes on an iBlot dry blotting system. Membranes were incubated with the rabbit polyclonal antibodies against TRAP, cathepsin K, MMP-9 and GAPDH at 4 °C overnight followed by incubation with goat-anti-rabbit alkaline-phosphatase-conjugated secondary antibody for 1 h. Blots were developed using iBlot Western Detection Chromogenic Kit and digital images of the blots were acquired using a flatbed scanner (Ricoh Aficio, Johannesburg, South Africa).

### 2.8. cDNA Synthesis and RT-PCR Analysis

RAW264.7 cells (4 × 10^4^ cells per well) were differentiated for 5 days in the presence of RANKL (15 ng·mL^−1^) and PUFAs (20 μg·mL^−1^) in a 24-well plate. Cell culture media and factors were exchanged after three days. Total cellular RNA was extracted from the cells using TRI^®^ reagent. One microgram of the extracted RNA was reverse transcribed into cDNA using M-MuLV reverse transcriptase and amplified through PCR using KAPA2G Robust HotStart ReadyMix as per manufacturer’s instructions. The primers (Inqaba Biotechnology, Pretoria, South Africa) used in this study are as follows: TRAP: Forward: CCACCCTGAGATTTGTGGCT, Reverse: ACATACCAGGGGATGTTGCG; cathepsin K: Forward: CTGGAGGGCCAACTCAAGA, Reverse: CCTCTGCATTTAGCTGCCTT; MMP9: Forward: GTCATCCAGTTTGGTGTCGCG, Reverse: AGGGGAAGACGCACAGCTC; GAPDH: Forward: GATGACATCAAGAAGGTGGTGAAGC, Reverse: ATACCAGGAAATGAGCTTGACAAA. PCR products were separated by electrophoresis on 1% agarose gels and visualised by ethidium bromide staining and documented using a Gel Documentation system attached to a monochrome scientific grade camera (E-Box 1000/26M, Vilber Lourmat, Cedex, France).

### 2.9. Statistics

Data are representative of three independent experiments with three replicates in each experiment unless otherwise stated and are expressed as means ± SEM (standard error of the mean). Statistical analysis was performed by two way analysis of variance (ANOVA) followed by Bonferroni post hoc test using GraphPad Prism software (GraphPad software Inc., La Jolla, CA, USA). *p*-Value < 0.05 was regarded as statistically significant.

## 3. Results

### 3.1. Effects of PUFAs on Osteoclastogenesis

**Figure 1:**
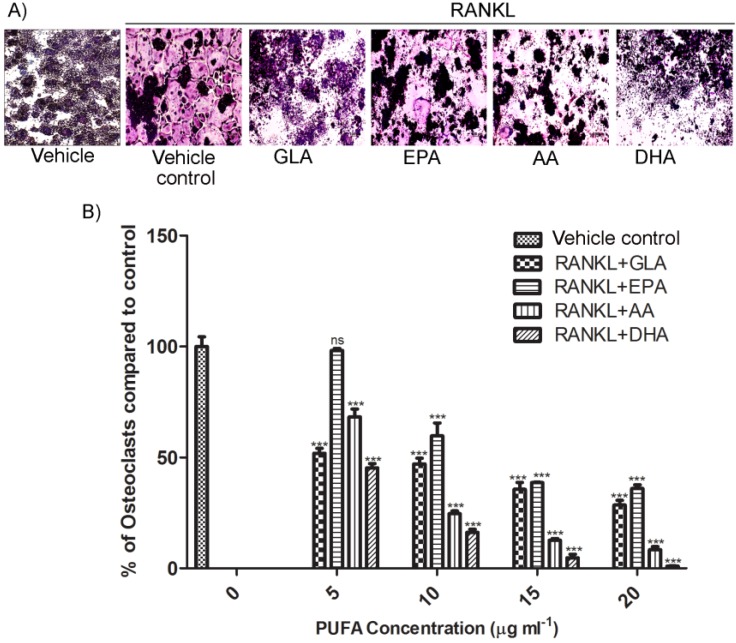
Effects of polyunsaturated fatty acids (PUFAs) on RANKL-stimulated osteoclast formation. (**A**) RAW264.7 murine macrophages cultured for 5 days with vehicle alone, with RANKL (15 ng·mL^−1^) or with various PUFAs: GLA, EPA, AA and DHA (20 μg·mL^−1^) in combination with RANKL are indicated. TRAP-positive cells that contain more than 3 nuclei were counted as osteoclasts. Osteoclasts stain purple-red in the presence of TRAP and appear as large multinucleated cells (MNCs). (**B**) The number of TRAP-positive MNCs as seen in (**A**) were counted in each well and expressed as percentage of TRAP-positive MNCs in the vehicle control. Statistical analysis was performed by two-way ANOVA followed by Bonferroni post hoc test. Three independent experiments were performed and each value represents the mean ± SEM of triplicate cultures; one representative field of view is shown for each exposure. (*** Significant difference from RANKL-control, *p* < 0.001; (*n* = 3), ns: no significance).

RAW264.7 macrophages were treated with or without 5–20 μg·mL^−1^ of each PUFA in the presence of RANKL to analyse osteoclast differentiation using TRAP staining (no cytotoxic effects observed at these concentrations, data not shown). Osteoclasts stain purple-red in the presence of TRAP and appear as large multinucleated cells. All the PUFAs inhibited RANKL-induced osteoclast differentiation (Figure 1A), however DHA and AA had the strongest inhibitory effect on TRAP-positive osteoclast formation and this occurred in a dose-dependent manner (Figure 1B) with DHA showing the most robust attenuation of osteoclastogenesis. Cells treated with PUFAs alone in the absence of RANKL did not show osteoclastogenic activity (data not shown). Since AA and DHA had the most significant inhibitory effect on osteoclastogenesis in our model, the effect of these PUFAs (representative of the ω6- and ω3-PUFA families) were investigated in subsequent experiments.

### 3.2. Effects of AA and DHA on Actin Ring Formation

**Figure 2:**
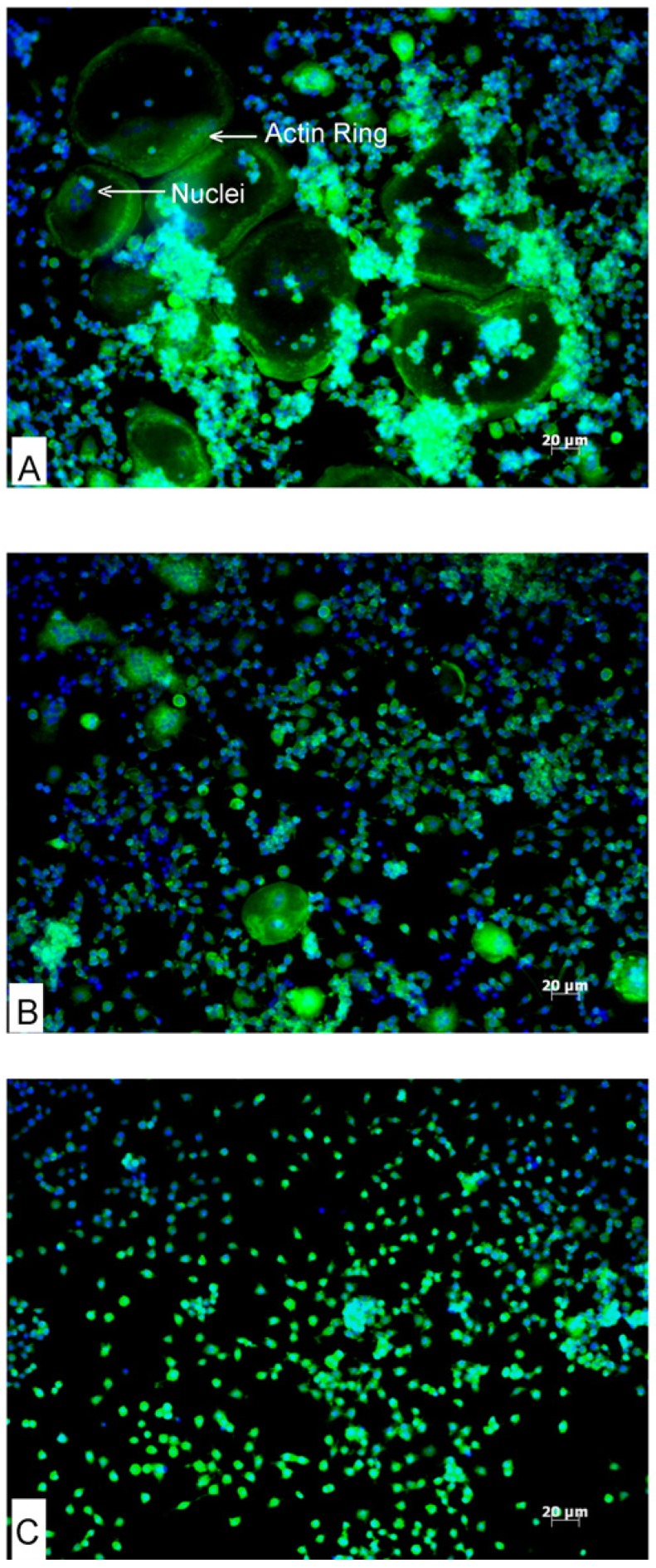
Effects of AA and DHA on RANKL-induced actin ring formation. RAW264.7 cells were differentiated into osteoclasts in the presence of 30 ng·mL^−1^ RANKL alone (**A**) or co-stimulated with AA (**B**), or DHA (**C**) at 20 μg·mL^−1^. Phalloidin (green fluorescence) stains the actin filaments, and Hoechst 33342 (blue) stains the nuclei of cells. Mature differentiated osteoclasts appear as large green cells with multiple nuclei and a green (actin) ring. Experiments were repeated three times in duplicate; one representative field of view is shown for each exposure.

Actin ring formation is an essential feature of osteoclast differentiation that is necessary for bone resorption. RAW264.7 cells were treated with RANKL to differentiate into osteoclasts and actin ring formation was visualised fluorescently (Figure 2). Following RANKL treatment, differentiated osteoclast-like cells were evenly distributed on the floor of the culture plate wells. These large osteoclast-like cells exhibited green actin ring cytoskeletal elements characteristic of osteoclasts (Figure 2A). These cells were also multinucleated (nuclei stained blue by Hoechst 33342). AA and DHA-treated cells did not differentiate into osteoclasts. Fewer and comparatively smaller actin rings were seen in cells treated with AA in combination with RANKL as compared to cells treated with RANKL alone; however actin rings were completely absent in cells exposed to DHA (Figure 2B,C).

### 3.3. Effects of AA and DHA on the Ultrastructure of Osteoclasts

**Figure 3:**
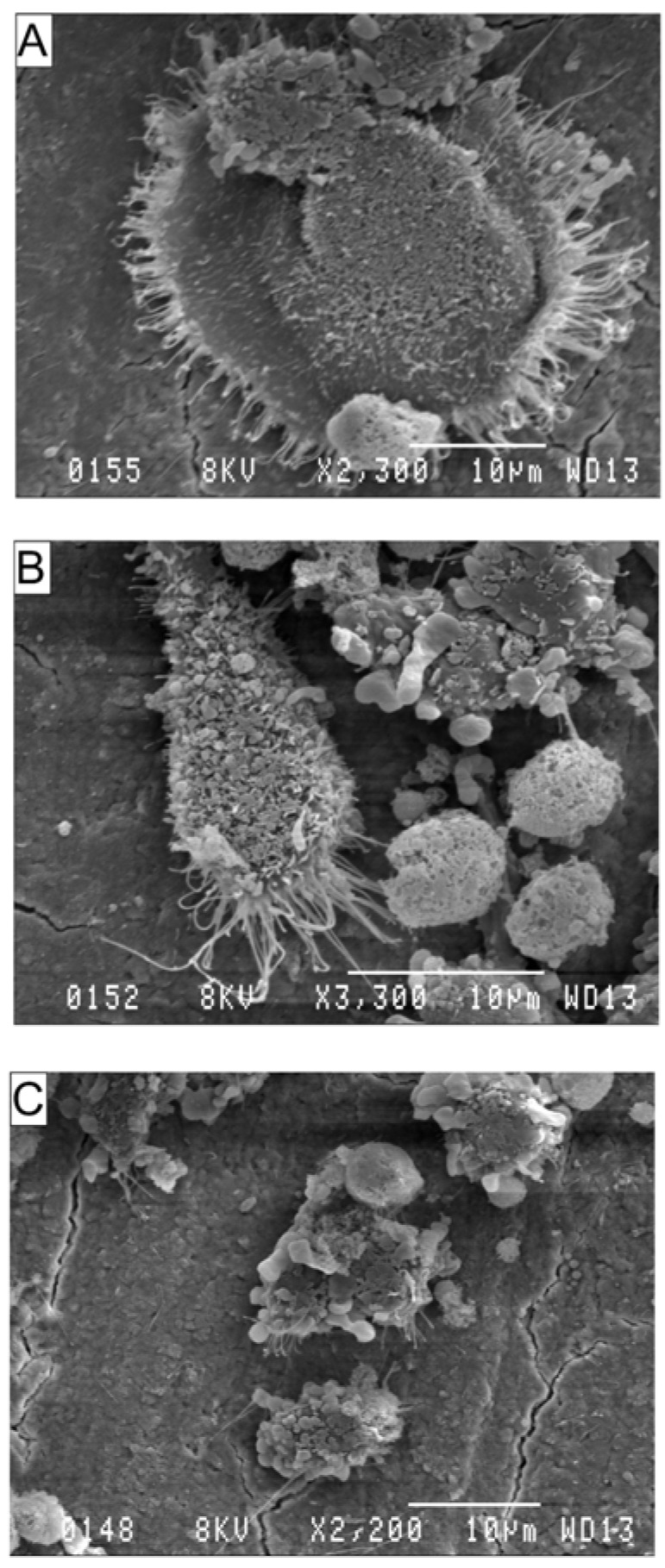
Effects of AA and DHA on osteoclast ultrastructure. Scanning electron micrographs (SEM) of RAW264.7 cells grown on bovine cortical bone slices. RAW264.7 cells were differentiated in the presence of 30 ng·mL^−1^ RANKL or PUFAs at 20 μg·mL^−1^ for 9 days as described in the Materials & Methods. RANKL (**A**), RANKL + AA (**B**) and RANKL + DHA (**C**). Experiments were repeated three times in duplicates; one representative field of view is shown for each exposure.

Structural surface changes that arise when pre-osteoclasts differentiate into mature bone resorbing osteoclasts can be seen in Figure 3. Figure 3A, shows a mature osteoclast differentiated from RAW264.7 cells in the presence of RANKL. This osteoclast clearly exhibits the ringed structure of F-actin-containing podosomes and ruffled borders, critical for osteoclast attachment to bone. RANKL-stimulated RAW264.7 cells treated with AA or DHA were unable to fully differentiate into mature osteoclasts and exhibited incomplete or absent podosome morphology (Figure 3B,C).

### 3.4. Effects of AA and DHA on Bone Resorption

**Figure 4:**
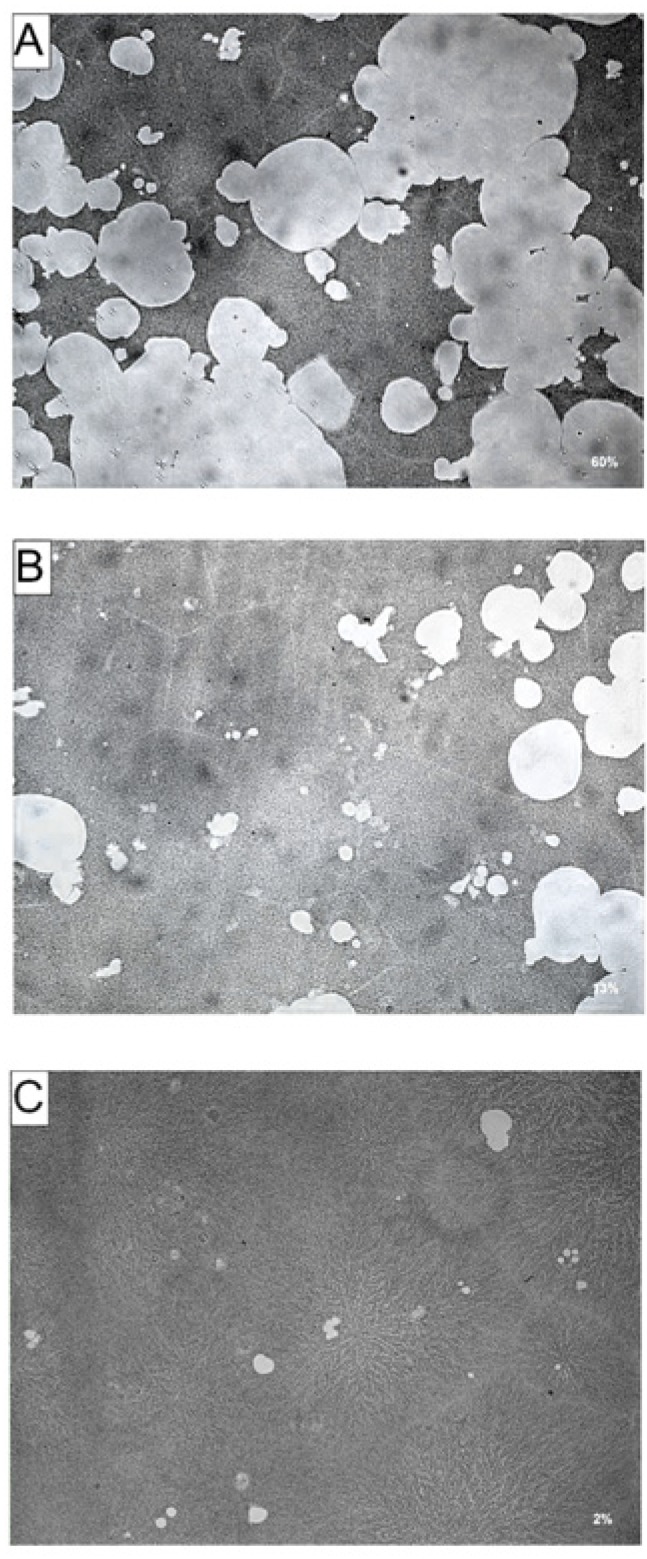
Effects of AA and DHA on resorption pit formation in Corning Osteo Assay surface plates. RAW264.7 cells were cultured in the presence of 30 ng·mL^−1^ RANKL (**A**) and AA (**B**) or DHA (**C**) at 20 μg·mL^−1^ on bone mimetic surface plates. After 7 days of culture the plates were washed in 5% sodium hypochlorite solution to remove the cells. The resorbed areas were then photographed with a digital camera attached to a microscope and the percentage resorption area per well was measured with ImageJ analysis software. (**A**) 60%, (**B**) 13%, (**C**) 2%. Experiments were repeated three times; one representative field of view is shown for each exposure.

To determine if the inhibitory effects of AA and DHA on RANKL-induced osteoclast formation results in reduced bone resorption, RAW264.7 cells were cultured on Corning Osteo-Assay plates, treated with AA or DHA in combination with RANKL for 9 days. Effects of attenuated osteoclast formation in cells exposed to AA or DHA were also seen in bone resorption experiments. RAW264.7 cultures exposed to AA (Figure 4B) or DHA (Figure 4C) showed a reduction in resorption area (white areas in photomicrographs) compared to RANKL-treated cells (Figure 4A). The total resorption area in wells exposed to AA or DHA at 20 μg·mL^−1^ were 13% and 2% respectively, significantly lower compared to RANKL stimulated cells, showing a resorption area of 60%.

### 3.5. Effects of AA and DHA on Osteoclast Specific Gene Expression

**Figure 5:**
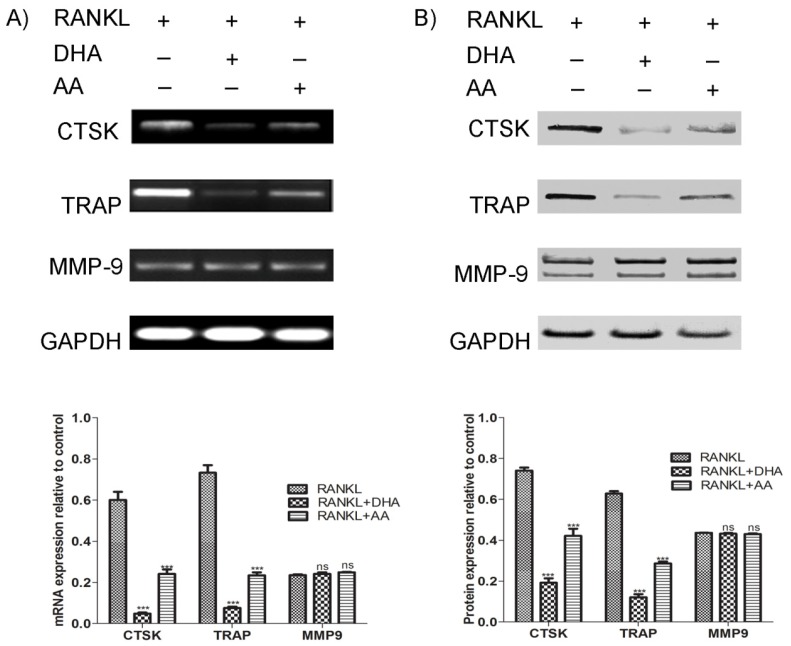
Effects of AA and DHA on RANKL-induced osteoclast specific gene expression. RAW264.7 cells were cultured in the presence of 15 ng·mL^−1^ RANKL and AA or DHA at 20 μg·mL^−1^ for 5 days, as indicated. (**A**) mRNA expression levels of cathepsin K (CTSK), tartrate resistant acid phosphatase (TRAP) and Matrix Metalloproteinase-9 (MMP-9) were examined using RT-PCR. Glyceraldehyde 3-phosphate dehydrogenase (GAPDH) served as loading control. (**B**) Protein expression levels of indicated osteoclast marker genes were analysed by western blotting. Band signal intensity was quantified by densitometry through NIH ImageJ software. The results were standardised against GAPDH levels and are presented as the relative density. Signal quantifications for each lane are shown as bar graphs. Statistical analysis was performed by two way ANOVA followed by Bonferroni post hoc test. The results are expressed as means ± SEM (*** *p* < 0.001; (*n* = 3), different from values after treatment with RANKL alone, ns: no significance). Experiments were repeated three times and the figures are representative of one experiment.

TRAP, cathepsin K and MMP-9 are highly expressed in osteoclastic cells and are considered as markers of the mature osteoclast. Therefore, we investigated the effects of AA and DHA on RANKL-induced osteoclast specific gene expression using RT-PCR and western blot analysis. As shown in Figure 5, treatment of AA or DHA in combination with RANKL suppressed mRNA- (Figure 5A) and protein expression levels (Figure 5B) of cathepsin K and TRAP but did not affect the expression of MMP-9 in comparison to RAW264.7 cells stimulated with RANKL alone. The DHA-mediated inhibitory effects on osteoclastogenic marker genes were stronger than AA.

## 4. Discussion

A balanced intake of ω3- and ω6-PUFAs could have advantageous effects on health, protecting and mitigating against the onset and development of cancer, and cardiovascular chronic diseases [18]. Several lines of evidence suggest that long chain polyunsaturated fatty acids (LCPUFAs) favor bone mass accrual [13]. Animals that receive inadequate dietary EFAs have been shown to develop severe osteoporosis, while the restoration of the ω3:ω6 ratio through adequate diet reversed the process of bone degradation [19]. Ovariectomized rats fed with safflower oil and/or high-oleate safflower oil blended with high DHA at a ω3-ω6 ratio of 5:1 experienced significantly lower bone loss, decreased serum concentration of bone resorption markers and increased concentration of bone formation markers [20].

Excessive osteoclast formation and bone resorption often occurs in response to increased RANKL activity that disrupts the coupling between bone formation and resorption as seen in a range of diseases such as osteoporosis, Paget’s disease, rheumatoid arthritis, periodontitis and bone metastases [21]. The attenuation of RANKL-induced osteoclast formation and bone resorption is one approach used to counteract pathological bone loss. Bioactive compounds that can abrogate RANKL-induced osteoclast formation with minimal side effects are of great biomedical interest. Using an *in vitro* model, we selected two ω3-PUFAs: EPA and DHA, and two ω6-PUFAs: AA and GLA and analysed their effects on osteoclastogenesis induced by RANKL using RAW264.7 macrophages. Although all of the PUFAs showed dose-dependent inhibitory effects on osteoclastogenesis, the degree to which they attenuated osteoclast formation varied widely at different concentrations. Among the tested PUFAs, at a concentration of 20 μg·mL^−1^ DHA and AA showed the strongest inhibitory effects. DHA-mediated inhibition however was more pronounced than that of AA. Since DHA and AA were the most potent of all the PUFAs at a particular concentration studied in our model we selected these two for further experiments. Circulating levels of free fatty acids in serum reported elsewhere are comparable with those studied in the present experiments [22].

The active osteoclast forms F-actin rings that are an essential cytoskeletal element necessary for the bone resorption process; the appearance of the actin ring is considered a functional marker for osteoclasts [23]. Prospective compounds with anti-osteoclastogenic activity and the potential to disrupt actin ring formation and cytoskeletal elements associated with bone resorption, could be used for treating bone loss related diseases. In our study, DHA and AA both inhibited osteoclastogenesis and this effect correlated with smaller and fewer actin rings in AA-treated cells and none in DHA-treated cells as compared to cells treated with RANKL alone. These results corroborate with the earlier reports showing the inhibitory effects of these PUFAs on TRAP-positive osteoclast formation [24]. Rapid cytoskeletal rearrangement occurs during osteoclast formation, with mature osteoclasts forming podosomes for attachment to the bone surface, and a ruffled border to act as a basolateral secretory membrane for solubilising the bone matrix [25]. Since, AA or DHA-treated cells were unable to differentiate into osteoclasts these cells did not exhibit podosomes or the ruffled border morphology compared to RANKL-treated control cells.

Lysosomal enzymes (TRAP and cathepsin K) and matrix metalloproteases such as MMP-9 play an important role in osteolysis. Targeted disruption of TRAP in mice resulted in reduced osteoclast activity [26]. Cathepsin K knockout mice experienced osteopetrosis (a congenital disorder in which the bones become overly dense) with severe loss in osteoclast activity [5]. The human disease pycnodysostosis that occurs due to mutations in cathepsin K gene have a phenotype similar with the knockout mice [27]. MMP9 also known as gelatinase B is expressed by active osteoclasts and giant tumors and plays an important role in resorption and tumor growth in the the bone micro-environment [28]. In the present study, AA and DHA both suppressed the RANKL-induced upregulation of TRAP and cathepsin K mRNA and protein expression; however, we did not find any change in MMP-9 expression. It has been reported that cathepsin K expression is crucial for actin ring formation and activation in osteoclasts [25]. Since, osteoclast formation was severely disrupted in AA or DHA-treated cells along with impaired cathepsin K and TRAP expression, cells in our model failed to form actin rings and exhibited none (DHA-treated) or disrupted (AA-treated) osteoclast structure. Mature multinucleated osteoclasts readily form actin ring structure, a process critical for bone resorption. This was reflected in our results by the significant increase in resorption area on the bone biomimetic material in the RANKL-treated control cells. However, DHA and AA impaired the osteoclast formation process and a significant reduction in resorption area was observed in cells treated with these PUFAs. Previous reports have shown that MMP-9 knockout in mice had no apparent loss in bone resorption although these mice had lengthened growth plates and experienced delayed osteoclast recruitment [29]. Osteoclasts express several other MMPs such as −1, −10, −12 and −14 (not analysed in this study) and it is possible that AA and DHA might act on other MMPs to inhibit osteoclast activity as their compensatory production has also been reported to play a role in osteoclast activity. Furthermore, other lines of evidence suggest that inhibition of MMP-9 does not result in complete inhibition of basal osteoclast activity *in vitro* supporting the aforementioned notion [30]. Additionally, cathepsins and MMPs appear to have similar functions in the bone resorption process and may be able to substitute for each other [31].

Previous studies have demonstrated the inhibitory effects of PUFAs on osteoclastogenesis; however, the differences in the *in vitro* models used as well as complex designs make it difficult to draw definitive conclusions on the precise effects of ω3- and ω6-PUFAs. A study on linoleic acid and conjugated linoleic acid revealed that these PUFAs have an inhibitory effect on the osteoclast formation of bone marrow macrophages and RAW264.7 cells [32]. In a subsequent study by the same group, DHA was found to be a more potent inhibitor of osteoclast differentiation than EPA in RAW264.7 cells [24], an observation in agreement with our results. In line with this, research conducted by Sun *et al.* (2003) reported the inhibitory effect of both EPA and DHA on osteoclastogenesis in bone marrow macrophages [33]. In a pilot study, Hutchins-Wiese *et al.* (2014) showed that high-dose EPA and DHA supplementation reduced bone resorption in postmenopausal breast cancer survivors on aromatase inhibitors [34]. Increased dietary intake of AA has also been shown to lower the risk of hip fractures by 80% in men [35]. In an another study, the researchers used fat-1 mouse, a transgenic model that synthesises ω3-PUFA from ω6-PUFAs and showed that the fat-1 ovariectomized mice had lower levels of RANKL and TRAP in serum and higher BMD than ovariectomized controls [36]. Microarray studies conducted by Akiyama *et al.* (2013) have reported the inhibitory effects of DHA and EPA on osteoclast specific genes [37].

PUFAs are incorporated into cell membranes where they modulate signaling processes, gene expression and functioning of cells [38]. The longer chain metabolites of LA and ALA, *i.e.*, GLA, AA, EPA and DHA are clinically important for various organs such as brain, kidney and liver [38]. Oxygenase enzymes such as cyclo-oxygenases and lipoxygenases metabolise PUFAs to produce short-lived biologically active compounds termed eicosanoids that control inflammatory and immune processes and might have effects on cardiovascular disease, blood pressure, and arthritis [39]. Although most of the eicosanoids are produced from AA; DGLA and EPA also serve as eicosanoid precursors [40]. Eicosanoids such as prostanoids (thromboxanes and prostaglandins) and leukotrienes are oxygenated derivatives of EFAs [41]. Other eicosonaids include lipoxins and resolvins. PUFAs may inhibit osteoclastogenesis through modulation of their metabolites and derivatives such as prostaglandins (PGs), leukotrienes and resolvins. Resolvin E1 (RvE1) a metabolite of EPA has been shown to inhibit osteoclast formation by interfering with the NFκ-B pathway and downregulation of DC-STAMP gene expression [42,43]. Yuan *et al.* by using bone marrow macrophages reported that DHA inhibited osteoclastogenesis via resolvin D1 (RvD1, a metabolite of DHA) and downregulation of DC-STAMP expression whereas DGLA, EPA and AA enhanced osteoclast formation through PGE_1_, PGE_2_ and PGE_3_ [44]. In our study we found that DHA, AA and EPA attenuated osteoclast formation with various degrees. The difference in Yuan *et al.* and our results may be due to the employment of different cell lines as well as the use of lower concentrations of various PUFAs analysed in the aforementioned study. Prostaglandins such as PGE_2_ and PGE_3_ metabolized via cyclooxygenase (COX) have been shown to regulate osteoclastogenesis both *in vitro* and *in vivo* [45]. Watkins *et al.* (2000) have reported the positive effects of lower dietary ω3-ω6 ratio on reduced PGE_2_ production in rats [46]. Lipoxin A_4_ a metabolite of AA has been reported to attenuate bone resorption and inhibit inflammation by downregulating pro-inflammatory cytokines [47]. PUFAs are also thought to inhibit the fusion of mononuclear osteoclast precursors by decreasing the production of nitrous oxide (NO) which occurs prior to pre-osteoclast fusion; this inhibition may prevent pre-osteoclast fusion [48]. More work is however needed to elucidate the cellular and molecular mechanisms of action of PUFAs and their metabolites on bone.

## 5. Conclusions

We have shown that the ω6-PUFA: AA as well as the ω3-PUFA: DHA potently inhibited the RANKL-mediated osteoclast formation from mononuclear precursors in the murine RAW264.7 macrophage model. The inhibitory effects of DHA and AA on osteoclast formation correlated with weakened osteoclast resorption and expression of osteoclast specific genes. DHA had the most potent inhibitory effect on osteoclastogenesis compared to AA, suggesting that optimal intake of these fatty acids in the diet could be helpful in maintaining bone mass, and inhibition of diseases characterised by excessive osteolysis.
